# (*Z*)-2-(4-*tert*-Butyl­phen­yl)-1-(4-chloro-1-ethyl-3-methyl-1*H*-pyrazol-5-yl)-2-cyano­vinyl pivalate

**DOI:** 10.1107/S1600536811027255

**Published:** 2011-07-13

**Authors:** Bao Wang, Haibo Yu, Bin Li

**Affiliations:** a Environmental Monitoring Station of Xinganmeng, Ulanhot 137400, People’s Republic of China; b State Key Laboratory of the Discovery and Development of Novel Pesticides, Shenyang Research Institute of Chemical Industry Co. Ltd, Shenyang 110021, People’s Republic of China

## Abstract

In the title compound, C_24_H_30_ClN_3_O_2_, the dihedral angle between the benzene and pyrazole rings is 56.86 (7)°. The C=C bond is significantly twisted, as indicated by the dihedral angle of 12.26 (1)° between the two sets of three atoms linked by the double bond.

## Related literature

The bioactivity of isomers of acrylonitrile compounds often differ, see: Kenzo *et al.* (2006 ▶); Yang *et al.* (2009 ▶).
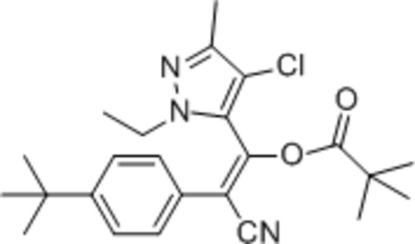


## Experimental

### 

#### Crystal data

C_24_H_30_ClN_3_O_2_
*M* *_r_* = 427.96
Monoclinic, 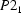

*a* = 10.1796 (7) Å
*b* = 10.5648 (7) Å
*c* = 12.3632 (8) Å
β = 110.613 (1)°
*V* = 1244.48 (14) Å^3^
*Z* = 2
Mo *K*α radiation
μ = 0.18 mm^−1^
*T* = 296 K
0.38 × 0.34 × 0.28 mm

#### Data collection

Bruker SMART CCD diffractometer
Absorption correction: multi-scan (*SADABS*; Bruker, 2001 ▶) *T*_min_ = 0.757, *T*_max_ = 1.000
6437 measured reflections
4354 independent reflections
3756 reflections with *I* > 2σ(*I*)
*R*_int_ = 0.014

#### Refinement

*R*[*F* ^2^ > 2σ(*F* ^2^)] = 0.039
*wR*(*F* ^2^) = 0.100
*S* = 1.03
4354 reflections
276 parameters
1 restraint
H-atom parameters constrained
Δρ_max_ = 0.14 e Å^−3^
Δρ_min_ = −0.17 e Å^−3^
Absolute structure: Flack (1983 ▶), 2014 Friedel pairs
Flack parameter: 0.06 (6)

### 

Data collection: *SMART* (Bruker, 2001 ▶); cell refinement: *SAINT* (Bruker, 2001 ▶); data reduction: *SAINT*; program(s) used to solve structure: *SHELXS97* (Sheldrick, 2008 ▶); program(s) used to refine structure: *SHELXL97* (Sheldrick, 2008 ▶); molecular graphics: *SHELXTL* (Sheldrick, 2008 ▶); software used to prepare material for publication: *SHELXTL* and *PLATON* (Spek, 2009 ▶).

## Supplementary Material

Crystal structure: contains datablock(s) I, global. DOI: 10.1107/S1600536811027255/hb5916sup1.cif

Structure factors: contains datablock(s) I. DOI: 10.1107/S1600536811027255/hb5916Isup2.hkl

Supplementary material file. DOI: 10.1107/S1600536811027255/hb5916Isup3.cml

Additional supplementary materials:  crystallographic information; 3D view; checkCIF report

## Figures and Tables

**Fig. 1.**
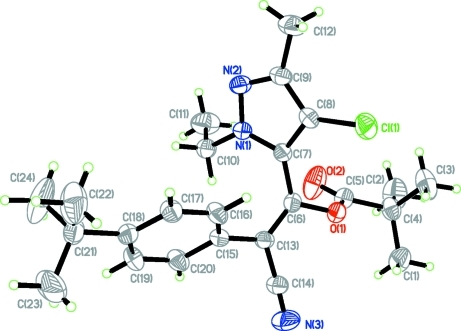
The molecular structure of (I), with 30% probability displacement ellipsoids.

**Fig. 2.**
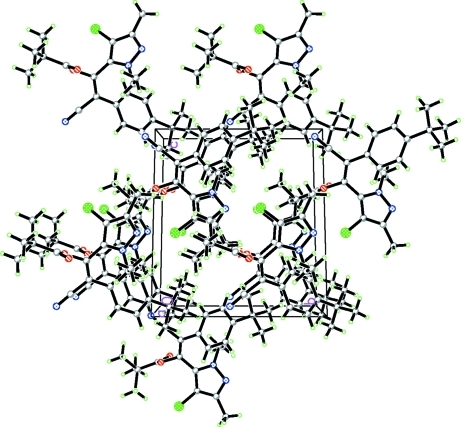
Crystal packing of (I).

## supplementary crystallographic information

### Comment

Acrylonitrile compounds display a broad range of biological, medical and pharmacological properties. There is a double bond in the molecule of the acrylonitrile compounds, and both geometric isomers referred to as the E- and *Z*-isomer can be present. The bioactivities of them often differ from each other (Kenzo *et al.*, 2006; Yang *et al.* 2009). In the process of preparation of the title compound, its geometric isomer product was also afforded, which showed obviously different acaricidal activity with the title compound. In order to confirm the geometry configuration, we report the crystal structure of the title compound (I) in this paper. The molecular structure of (I) is shown in Fig. 1. The the benzene and pyrazole rings in each of the ligands are not coplanar, the dihedral angle formed by the least-squares planes of the benzene and pyrazole rings being equal to 56.86 (7)°. The dihedral angle between C7/C6/O1 and C15/C13/C14 is 12.26 (1)°. The C(14)—C(13)—C(15)—C(20), O(1)—C(6)—C(13)—C(14), C(7)—C(6)—C(13)—C(14) and C(5)—O(1)—C(6)—C(7) torsion angles are -44.9 (3), -13.6 (3), 172.1 (2) and -69.5 (2)°, respectively. The crystal packing of (I) shows in Fig. 2. No significant interactions, such as hydrogen bonds or pi-pi stacking, are observed in (I). Examination of this structure with *PLATON*(Spek, 2009) reveals no solvent-accessible voids in the unit cell.

### Experimental

The title compound was synthesized by 2-(4-(*tert*-butyl) phenyl)-3-(4-chloro-1-ethyl-3-methyl-1*H*-pyrazol-5-yl) -3-hydroxyacrylonitrile with pivaloyl chloride in THF. The crude products were purified by silica-gel column chromatography and then grown from heptane to afford colorless blocks of (I). To the mixture of 2-(4-(*tert*-butyl)phenyl)-3-(4-chloro- 1-ethyl-3-methyl-1*H*-pyrazol-5-yl)-3-hydroxyacrylonitrile (0.69 g, 2.0 mmol) and triethyl amine (0.24 g, 2.4 mmol) in THF (10 ml), pivaloyl chloride (0.29 g, 2.4 mmol) was added dropwise at roomtemperature and reacted for 1 h. After separation through silica gel column chromatography (fluent: ethyl acetate/petroleum ether=1/20), The title product compound was gained as a white solid (0.13 g, 15%).

Anal. Calcd for C_24_H_30_Cl_1_N_3_O_2_: C, 67.35; H, 7.07; Cl, 8.28; N, 9.82; O, 7.48. Found: C, 67.33; H, 7.11; N, Cl, 8.32; N, 9.85; O, 7.52. ^1^H NMR(DMSO): 0.98 (s, 9H, CO(CH_3_)_3_), 1.27 (s, 9H, Ph-(CH_3_)_3_), 1.36 (t, 3H, CH_3_), 2.25 (s, 3H, CH_3_), 3.60 (q, 2H, N—CH_2_), 7.06 (d, 2H, Ph), 7.31 (d, 2H, Ph).

### Refinement

Although all H atoms were visible in difference maps, they werefinally placed in geometrically calculated positions, with C—H distances in the range 0.93–0.97 Å, and included in the final refinement in the riding model approximation, with *U*_iso_(H) = 1.2*U*_eq_(C) and *U*_iso_(H) = 1.5*U*_eq_(C).

### Crystal data

**Table:**

C_24_H_30_ClN_3_O_2_	*F*(000) = 456
*M**_r_* = 427.96	*D*_x_ = 1.142 Mg m^−^^3^
Monoclinic, *P*2_1_	Mo *K*α radiation, λ = 0.71073 Å
*a* = 10.1796 (7) Å	Cell parameters from 2837 reflections
*b* = 10.5648 (7) Å	θ = 2.6–22.6°
*c* = 12.3632 (8) Å	µ = 0.18 mm^−^^1^
β = 110.613 (1)°	*T* = 296 K
*V* = 1244.48 (14) Å^3^	Block, colorless
*Z* = 2	0.38 × 0.34 × 0.28 mm

### Data collection

**Table:**

Bruker SMART CCD diffractometer	4354 independent reflections
Radiation source: fine-focus sealed tube	3756 reflections with *I* > 2σ(*I*)
graphite	*R*_int_ = 0.014
phi and ω scans	θ_max_ = 25.0°, θ_min_ = 1.8°
Absorption correction: multi-scan (*SADABS*; Bruker, 2001)	*h* = −12→11
*T*_min_ = 0.757, *T*_max_ = 1.000	*k* = −12→12
6437 measured reflections	*l* = −7→14

### Refinement

**Table:**

Refinement on *F*^2^	Secondary atom site location: difference Fourier map
Least-squares matrix: full	Hydrogen site location: inferred from neighbouring sites
*R*[*F*^2^ > 2σ(*F*^2^)] = 0.039	H-atom parameters constrained
*wR*(*F*^2^) = 0.100	*w* = 1/[σ^2^(*F*_o_^2^) + (0.0554*P*)^2^ + 0.0882*P*] where *P* = (*F*_o_^2^ + 2*F*_c_^2^)/3
*S* = 1.03	(Δ/σ)_max_ < 0.001
4354 reflections	Δρ_max_ = 0.14 e Å^−^^3^
276 parameters	Δρ_min_ = −0.17 e Å^−^^3^
1 restraint	Absolute structure: Flack (1983), 2014 Friedel pairs
Primary atom site location: structure-invariant direct methods	Flack parameter: 0.06 (6)

### Special details

**Table:**

Geometry. All e.s.d.'s (except the e.s.d. in the dihedral angle between two l.s. planes) are estimated using the full covariance matrix. The cell e.s.d.'s are taken into account individually in the estimation of e.s.d.'s in distances, angles and torsion angles; correlations between e.s.d.'s in cell parameters are only used when they are defined by crystal symmetry. An approximate (isotropic) treatment of cell e.s.d.'s is used for estimating e.s.d.'s involving l.s. planes.
Refinement. Refinement of *F*^2^ against ALL reflections. The weighted *R*-factor *wR* and goodness of fit *S* are based on *F*^2^, conventional *R*-factors *R* are based on *F*, with *F* set to zero for negative *F*^2^. The threshold expression of *F*^2^ > σ(*F*^2^) is used only for calculating *R*-factors(gt) *etc*. and is not relevant to the choice of reflections for refinement. *R*-factors based on *F*^2^ are statistically about twice as large as those based on *F*, and *R*- factors based on ALL data will be even larger.

### Fractional atomic coordinates and isotropic or equivalent isotropic displacement parameters (Å^2^)

**Table:**

	*x*	*y*	*z*	*U*_iso_*/*U*_eq_	
Cl1	0.91735 (8)	0.12628 (7)	0.43386 (5)	0.0805 (2)	
O1	0.88635 (15)	0.00475 (13)	0.67430 (13)	0.0546 (4)	
O2	0.6841 (2)	0.0528 (2)	0.6923 (2)	0.1073 (8)	
N1	0.83545 (19)	0.33952 (17)	0.65433 (15)	0.0533 (4)	
N2	0.7959 (2)	0.41956 (19)	0.56420 (17)	0.0611 (5)	
N3	1.0994 (3)	−0.0527 (2)	0.9522 (2)	0.0910 (8)	
C1	0.8112 (4)	−0.2484 (3)	0.6992 (3)	0.0988 (10)	
H1A	0.9057	−0.2303	0.7043	0.148*	
H1B	0.7866	−0.3327	0.6700	0.148*	
H1C	0.8045	−0.2418	0.7745	0.148*	
C2	0.5602 (4)	−0.1789 (4)	0.6095 (4)	0.1147 (13)	
H2A	0.5559	−0.1802	0.6859	0.172*	
H2B	0.5301	−0.2592	0.5729	0.172*	
H2C	0.4999	−0.1133	0.5650	0.172*	
C3	0.7257 (4)	−0.1646 (4)	0.4991 (3)	0.1048 (11)	
H3A	0.6715	−0.0995	0.4489	0.157*	
H3B	0.6926	−0.2461	0.4664	0.157*	
H3C	0.8227	−0.1554	0.5077	0.157*	
C4	0.7104 (3)	−0.1533 (3)	0.6172 (2)	0.0666 (6)	
C5	0.7515 (2)	−0.0214 (2)	0.6629 (2)	0.0581 (6)	
C6	0.9388 (2)	0.1258 (2)	0.70985 (16)	0.0475 (4)	
C7	0.8815 (2)	0.2278 (2)	0.62748 (17)	0.0483 (5)	
C8	0.8687 (2)	0.2379 (2)	0.51326 (17)	0.0532 (5)	
C9	0.8173 (2)	0.3588 (2)	0.47794 (19)	0.0567 (5)	
C10	0.8154 (2)	0.3768 (2)	0.76083 (17)	0.0670 (6)	
H10A	0.8685	0.4533	0.7904	0.080*	
H10B	0.8513	0.3107	0.8182	0.080*	
C11	0.6638 (2)	0.3997 (2)	0.74279 (17)	0.1022 (11)	
H11A	0.6289	0.4674	0.6884	0.153*	
H11B	0.6547	0.4223	0.8150	0.153*	
H11C	0.6109	0.3242	0.7134	0.153*	
C12	0.7861 (3)	0.4190 (3)	0.3620 (2)	0.0822 (8)	
H12A	0.7061	0.3785	0.3067	0.123*	
H12B	0.8658	0.4099	0.3382	0.123*	
H12C	0.7663	0.5073	0.3667	0.123*	
C13	1.0443 (2)	0.1363 (2)	0.81182 (16)	0.0479 (4)	
C14	1.0747 (2)	0.0294 (2)	0.8883 (2)	0.0589 (6)	
C15	1.1321 (2)	0.2504 (2)	0.85119 (16)	0.0481 (5)	
C16	1.1890 (2)	0.3123 (2)	0.78031 (18)	0.0578 (6)	
H16	1.1715	0.2823	0.7058	0.069*	
C17	1.2721 (2)	0.4187 (2)	0.81808 (19)	0.0583 (5)	
H17	1.3087	0.4590	0.7681	0.070*	
C18	1.3018 (2)	0.4663 (2)	0.9282 (2)	0.0543 (5)	
C19	1.2453 (3)	0.4019 (3)	0.9993 (2)	0.0660 (6)	
H19	1.2629	0.4315	1.0739	0.079*	
C20	1.1640 (3)	0.2955 (2)	0.96248 (19)	0.0630 (6)	
H20	1.1299	0.2533	1.0132	0.076*	
C21	1.3905 (3)	0.5854 (2)	0.9708 (2)	0.0702 (7)	
C22	1.4498 (5)	0.6366 (4)	0.8833 (4)	0.1287 (15)	
H22A	1.5104	0.5744	0.8688	0.193*	
H22B	1.5023	0.7123	0.9130	0.193*	
H22C	1.3743	0.6556	0.8126	0.193*	
C23	1.5085 (5)	0.5570 (5)	1.0820 (4)	0.1517 (19)	
H23A	1.5733	0.4995	1.0673	0.227*	
H23B	1.4714	0.5193	1.1358	0.227*	
H23C	1.5563	0.6342	1.1140	0.227*	
C24	1.2955 (5)	0.6859 (4)	0.9907 (6)	0.170 (2)	
H24A	1.3499	0.7595	1.0241	0.255*	
H24B	1.2515	0.6538	1.0422	0.255*	
H24C	1.2247	0.7081	0.9183	0.255*	

### Atomic displacement parameters (Å^2^)

**Table:**

	*U*^11^	*U*^22^	*U*^33^	*U*^12^	*U*^13^	*U*^23^
Cl1	0.1082 (5)	0.0814 (4)	0.0610 (3)	0.0026 (4)	0.0413 (3)	−0.0081 (3)
O1	0.0532 (8)	0.0480 (8)	0.0624 (9)	−0.0004 (6)	0.0201 (7)	−0.0052 (7)
O2	0.0921 (14)	0.0818 (14)	0.178 (2)	−0.0117 (12)	0.0853 (16)	−0.0348 (15)
N1	0.0636 (11)	0.0477 (10)	0.0436 (9)	0.0047 (9)	0.0125 (8)	0.0029 (8)
N2	0.0659 (12)	0.0518 (11)	0.0584 (11)	0.0022 (9)	0.0130 (9)	0.0111 (9)
N3	0.1001 (17)	0.0791 (16)	0.0874 (17)	0.0104 (14)	0.0250 (14)	0.0343 (15)
C1	0.132 (3)	0.0590 (17)	0.102 (2)	−0.0037 (18)	0.037 (2)	−0.0024 (16)
C2	0.092 (2)	0.113 (3)	0.154 (3)	−0.044 (2)	0.062 (2)	−0.035 (3)
C3	0.136 (3)	0.105 (2)	0.081 (2)	−0.043 (2)	0.0475 (19)	−0.0326 (19)
C4	0.0687 (15)	0.0691 (15)	0.0645 (14)	−0.0174 (13)	0.0267 (12)	−0.0106 (13)
C5	0.0579 (13)	0.0565 (13)	0.0620 (13)	−0.0018 (11)	0.0237 (11)	−0.0025 (11)
C6	0.0525 (11)	0.0435 (10)	0.0486 (10)	−0.0008 (10)	0.0205 (9)	−0.0027 (10)
C7	0.0492 (11)	0.0481 (12)	0.0437 (11)	−0.0029 (9)	0.0116 (8)	−0.0015 (9)
C8	0.0534 (12)	0.0613 (14)	0.0425 (11)	−0.0060 (10)	0.0139 (9)	−0.0029 (10)
C9	0.0533 (12)	0.0623 (14)	0.0474 (12)	−0.0064 (10)	0.0089 (9)	0.0106 (11)
C10	0.0819 (16)	0.0622 (14)	0.0536 (13)	0.0178 (12)	0.0196 (11)	−0.0029 (11)
C11	0.101 (2)	0.129 (3)	0.088 (2)	0.046 (2)	0.0465 (17)	0.020 (2)
C12	0.0894 (18)	0.089 (2)	0.0608 (15)	−0.0074 (16)	0.0171 (13)	0.0255 (14)
C13	0.0493 (10)	0.0498 (11)	0.0432 (10)	0.0054 (10)	0.0146 (8)	0.0046 (9)
C14	0.0578 (13)	0.0611 (14)	0.0535 (12)	0.0024 (11)	0.0144 (10)	0.0082 (12)
C15	0.0473 (10)	0.0496 (12)	0.0435 (11)	0.0035 (9)	0.0113 (8)	0.0036 (9)
C16	0.0646 (13)	0.0678 (15)	0.0399 (11)	−0.0063 (11)	0.0173 (9)	−0.0044 (10)
C17	0.0624 (13)	0.0601 (14)	0.0532 (13)	−0.0079 (11)	0.0211 (10)	0.0024 (11)
C18	0.0503 (11)	0.0492 (12)	0.0581 (13)	0.0035 (9)	0.0122 (9)	−0.0026 (10)
C19	0.0712 (14)	0.0778 (17)	0.0463 (12)	−0.0073 (13)	0.0172 (11)	−0.0142 (12)
C20	0.0714 (14)	0.0747 (17)	0.0433 (12)	−0.0100 (13)	0.0207 (10)	0.0018 (11)
C21	0.0713 (15)	0.0530 (14)	0.0815 (17)	−0.0066 (11)	0.0209 (13)	−0.0120 (12)
C22	0.160 (4)	0.104 (3)	0.139 (3)	−0.067 (3)	0.073 (3)	−0.032 (3)
C23	0.127 (3)	0.126 (3)	0.136 (3)	−0.064 (3)	−0.035 (3)	0.008 (3)
C24	0.157 (4)	0.074 (2)	0.299 (7)	−0.007 (3)	0.107 (5)	−0.066 (4)

### Geometric parameters (Å, °)

**Table:**

Cl1—C8	1.715 (2)	C11—H11B	0.9600
O1—C5	1.358 (3)	C11—H11C	0.9600
O1—C6	1.396 (3)	C12—H12A	0.9600
O2—C5	1.181 (3)	C12—H12B	0.9600
N1—N2	1.343 (2)	C12—H12C	0.9600
N1—C7	1.354 (3)	C13—C14	1.434 (3)
N1—C10	1.456 (3)	C13—C15	1.478 (3)
N2—C9	1.327 (3)	C15—C16	1.374 (3)
N3—C14	1.140 (3)	C15—C20	1.382 (3)
C1—C4	1.536 (4)	C16—C17	1.385 (3)
C1—H1A	0.9600	C16—H16	0.9300
C1—H1B	0.9600	C17—C18	1.382 (3)
C1—H1C	0.9600	C17—H17	0.9300
C2—C4	1.522 (4)	C18—C19	1.386 (3)
C2—H2A	0.9600	C18—C21	1.530 (3)
C2—H2B	0.9600	C19—C20	1.375 (3)
C2—H2C	0.9600	C19—H19	0.9300
C3—C4	1.527 (4)	C20—H20	0.9300
C3—H3A	0.9600	C21—C23	1.504 (5)
C3—H3B	0.9600	C21—C22	1.512 (5)
C3—H3C	0.9600	C21—C24	1.513 (5)
C4—C5	1.506 (4)	C22—H22A	0.9600
C6—C13	1.342 (3)	C22—H22B	0.9600
C6—C7	1.456 (3)	C22—H22C	0.9600
C7—C8	1.376 (3)	C23—H23A	0.9600
C8—C9	1.391 (3)	C23—H23B	0.9600
C9—C12	1.496 (3)	C23—H23C	0.9600
C10—C11	1.4985	C24—H24A	0.9600
C10—H10A	0.9700	C24—H24B	0.9600
C10—H10B	0.9700	C24—H24C	0.9600
C11—H11A	0.9600		
			
C5—O1—C6	118.67 (16)	H11A—C11—H11C	109.5
N2—N1—C7	111.98 (17)	H11B—C11—H11C	109.5
N2—N1—C10	118.92 (17)	C9—C12—H12A	109.5
C7—N1—C10	128.93 (18)	C9—C12—H12B	109.5
C9—N2—N1	106.04 (18)	H12A—C12—H12B	109.5
C4—C1—H1A	109.5	C9—C12—H12C	109.5
C4—C1—H1B	109.5	H12A—C12—H12C	109.5
H1A—C1—H1B	109.5	H12B—C12—H12C	109.5
C4—C1—H1C	109.5	C6—C13—C14	118.0 (2)
H1A—C1—H1C	109.5	C6—C13—C15	124.60 (19)
H1B—C1—H1C	109.5	C14—C13—C15	117.39 (17)
C4—C2—H2A	109.5	N3—C14—C13	177.6 (3)
C4—C2—H2B	109.5	C16—C15—C20	117.8 (2)
H2A—C2—H2B	109.5	C16—C15—C13	121.33 (18)
C4—C2—H2C	109.5	C20—C15—C13	120.79 (19)
H2A—C2—H2C	109.5	C15—C16—C17	121.02 (19)
H2B—C2—H2C	109.5	C15—C16—H16	119.5
C4—C3—H3A	109.5	C17—C16—H16	119.5
C4—C3—H3B	109.5	C18—C17—C16	121.6 (2)
H3A—C3—H3B	109.5	C18—C17—H17	119.2
C4—C3—H3C	109.5	C16—C17—H17	119.2
H3A—C3—H3C	109.5	C17—C18—C19	116.8 (2)
H3B—C3—H3C	109.5	C17—C18—C21	122.5 (2)
C5—C4—C2	109.1 (2)	C19—C18—C21	120.8 (2)
C5—C4—C3	108.9 (2)	C20—C19—C18	121.8 (2)
C2—C4—C3	111.4 (3)	C20—C19—H19	119.1
C5—C4—C1	109.0 (2)	C18—C19—H19	119.1
C2—C4—C1	110.4 (3)	C19—C20—C15	120.9 (2)
C3—C4—C1	108.1 (3)	C19—C20—H20	119.5
O2—C5—O1	120.6 (2)	C15—C20—H20	119.5
O2—C5—C4	128.0 (2)	C23—C21—C22	109.5 (3)
O1—C5—C4	111.3 (2)	C23—C21—C24	110.0 (4)
C13—C6—O1	117.54 (19)	C22—C21—C24	107.7 (4)
C13—C6—C7	125.9 (2)	C23—C21—C18	109.6 (2)
O1—C6—C7	116.29 (16)	C22—C21—C18	112.6 (2)
N1—C7—C8	105.53 (18)	C24—C21—C18	107.4 (2)
N1—C7—C6	124.18 (18)	C21—C22—H22A	109.5
C8—C7—C6	130.2 (2)	C21—C22—H22B	109.5
C7—C8—C9	106.45 (19)	H22A—C22—H22B	109.5
C7—C8—Cl1	126.27 (18)	C21—C22—H22C	109.5
C9—C8—Cl1	127.17 (16)	H22A—C22—H22C	109.5
N2—C9—C8	109.98 (18)	H22B—C22—H22C	109.5
N2—C9—C12	121.6 (2)	C21—C23—H23A	109.5
C8—C9—C12	128.4 (2)	C21—C23—H23B	109.5
N1—C10—C11	111.96 (10)	H23A—C23—H23B	109.5
N1—C10—H10A	109.2	C21—C23—H23C	109.5
C11—C10—H10A	109.2	H23A—C23—H23C	109.5
N1—C10—H10B	109.2	H23B—C23—H23C	109.5
C11—C10—H10B	109.2	C21—C24—H24A	109.5
H10A—C10—H10B	107.9	C21—C24—H24B	109.5
C10—C11—H11A	109.5	H24A—C24—H24B	109.5
C10—C11—H11B	109.5	C21—C24—H24C	109.5
H11A—C11—H11B	109.5	H24A—C24—H24C	109.5
C10—C11—H11C	109.5	H24B—C24—H24C	109.5
			
C7—N1—N2—C9	−0.3 (2)	Cl1—C8—C9—C12	2.9 (3)
C10—N1—N2—C9	−175.99 (18)	N2—N1—C10—C11	60.59 (19)
C6—O1—C5—O2	−6.7 (3)	C7—N1—C10—C11	−114.27 (19)
C6—O1—C5—C4	176.59 (18)	O1—C6—C13—C14	−13.6 (3)
C2—C4—C5—O2	1.9 (4)	C7—C6—C13—C14	172.1 (2)
C3—C4—C5—O2	123.7 (3)	O1—C6—C13—C15	166.20 (18)
C1—C4—C5—O2	−118.6 (3)	C7—C6—C13—C15	−8.1 (3)
C2—C4—C5—O1	178.4 (2)	C6—C13—C14—N3	−137 (6)
C3—C4—C5—O1	−59.9 (3)	C15—C13—C14—N3	43 (7)
C1—C4—C5—O1	57.8 (3)	C6—C13—C15—C16	−47.2 (3)
C5—O1—C6—C13	115.7 (2)	C14—C13—C15—C16	132.6 (2)
C5—O1—C6—C7	−69.5 (2)	C6—C13—C15—C20	135.3 (2)
N2—N1—C7—C8	−0.6 (2)	C14—C13—C15—C20	−44.9 (3)
C10—N1—C7—C8	174.54 (19)	C20—C15—C16—C17	−1.9 (3)
N2—N1—C7—C6	176.18 (18)	C13—C15—C16—C17	−179.5 (2)
C10—N1—C7—C6	−8.7 (3)	C15—C16—C17—C18	0.4 (3)
C13—C6—C7—N1	−53.3 (3)	C16—C17—C18—C19	0.4 (3)
O1—C6—C7—N1	132.4 (2)	C16—C17—C18—C21	−178.5 (2)
C13—C6—C7—C8	122.7 (2)	C17—C18—C19—C20	0.2 (4)
O1—C6—C7—C8	−51.6 (3)	C21—C18—C19—C20	179.2 (2)
N1—C7—C8—C9	1.2 (2)	C18—C19—C20—C15	−1.8 (4)
C6—C7—C8—C9	−175.3 (2)	C16—C15—C20—C19	2.6 (3)
N1—C7—C8—Cl1	177.54 (16)	C13—C15—C20—C19	−179.8 (2)
C6—C7—C8—Cl1	1.0 (3)	C17—C18—C21—C23	−126.8 (4)
N1—N2—C9—C8	1.1 (2)	C19—C18—C21—C23	54.3 (4)
N1—N2—C9—C12	−179.5 (2)	C17—C18—C21—C22	−4.7 (4)
C7—C8—C9—N2	−1.5 (2)	C19—C18—C21—C22	176.4 (3)
Cl1—C8—C9—N2	−177.76 (17)	C17—C18—C21—C24	113.7 (4)
C7—C8—C9—C12	179.2 (2)	C19—C18—C21—C24	−65.2 (4)
